# Tetracycline residues in fresh dairy milk from three districts in Indonesia: Occurrence and dietary exposure assessment

**DOI:** 10.14202/vetworld.2023.2230-2235

**Published:** 2023-11-02

**Authors:** Raphaella Widiastuti, Eny Martindah, Yessy Anastasia

**Affiliations:** 1 Research Center for Veterinary Science, The National Research and Innovation Agency, Cibinong Science Center, Jl. Raya Jakarta-Bogor KM. 46, Cibinong, Kab. Bogor 16911, Indonesia; 2 Indonesian Research Center for Veterinary Science, Ministry of Agriculture, Jl. RE Martadinata 30, Bogor 16114, Indonesia

**Keywords:** dietary exposure assessment, fresh dairy milk, high-performance liquid chromatography, residues, tetracycline

## Abstract

**Background and Aim:**

Milk can introduce antibiotics into the human diet which poses a public health risk. Therefore, a study to determine the tetracycline residue in dairy milk and its health risk assessment is needed. A cross-sectional study was performed to detect tetracycline residues in fresh dairy milk samples collected from the districts of Malang, Boyolali, and Padang Panjang, Indonesia, and to evaluate dietary exposure to tetracycline residues through milk consumption in 10–12-year-old children and adults.

**Materials and Methods:**

A total of 203 fresh dairy milk samples were collected from local and smallholder dairy cows in Malang, Boyolali, and Padang Panjang in April and August 2018. High-performance liquid chromatography equipped with a photodiode array at 355 and 368 nm was used to detect tetracycline residues. Data were evaluated for dietary exposure assessment.

**Results:**

The results showed that the most common residue found was chlortetracycline (8.37%), followed by tetracycline (7.88%) and oxytetracycline (5.91%) in the concentration range of 14.8–498.4, 11.7–49.4, and 11.6–85.6 ng/g, respectively. Seven (3.45%) samples exceeded the maximum residue limit (MRL) for chlortetracycline. However, neither oxytetracycline nor tetracycline residues exceeded the MRL. The mean concentration of the tetracycline residues was 21.76–137.05 ng/g, resulting in an estimated daily intake of 16.46–172.83 ng/kg body weight/day.

**Conclusion:**

Tetracycline residues were found in almost all milk sampling locations. The highest prevalence and residue concentration were obtained from chlortetracycline. Estimated daily intake of tetracycline through milk by 10–12-year-old children and adult consumers was low and the risk to consumers was negligible.

## Introduction

Milk is one of the most nutritionally complete diets for human growth. It supplies substantial amounts of necessary nutrients. However, milk can introduce antibiotics into the human diet, posing a public health risk. Antibiotics are increasingly used to improve the quality and amount of milk produced and to prevent or treat animal infections [1]. Tetracyclines are broad-spectrum veterinary drugs commonly used in dairy farming to treat mastitis during lactation [2] and for worldwide prophylaxis and growth promotion. Tetracyclines can induce allergic responses, bacterial resistance, teratogenicity, and teeth discoloration in newborns and children >12 years of age when used during the first trimester of pregnancy [3]. Antibiotic residues in milk may inhibit the normal microflora and interfere with the manufacturing processes of some dairy products, such as yogurt and cheese [4].

Due to their impact on human health, a maximum residue limit (MRL) of tetracycline in milk has been established at 100 ng/g in many countries. The Joint FAO/WHO Expert Committee on Food Additives (JECFA) determined that 30 μg/kg body weight (BW)/day is the acceptable daily intake (ADI) for oxytetracycline residues [5]. On January 01, 2018, Indonesia prohibited the distribution or use of veterinary drugs unless they are used for medicinal purposes, as regulated by the Minister of Agriculture of the Republic of Indonesia through 14/PERMENTAN/PK.350/5/2017 concerning the classification of veterinary drugs.

Indonesia remains the lowest milk-consuming nation among the Association of Southeast Asian Nations, with an estimated country-specific intake of <30 kg or 29.1 L per capita/year [6]. However, urban households, with higher household incomes and children aged <12 years old, tend to consume milk more routinely than rural households [7]. Therefore, the risk of consuming food products with drug residues has become more widely recognized. Risk assessment provides an understanding of the relationship between reducing consumer risk and food risk from adverse human health effects, to ensure food safety. The lack of information regarding the long-term bioaccumulation of these substances raises concerns about the risk of drug residues in food.

Studies on the presence of antibiotic residues, including tetracyclines, in milk have been scarce in Indonesia. Reports on dietary exposure assessment to estimate the risk of tetracycline residues in milk are lacking. Tetracycline is the most commonly used antibiotic in dairy cattle in Indonesia [8]. Oxytetracycline residues were detected by high-performance liquid chromatography (HPLC) in 4/24 dairy milk samples taken from a sub-district of Malang, at concentrations ranging from 6.67 ng/g to 10.19 ng/g [9]. Oxytetracyclines were detected using the bioassay method in 1/15 samples of fresh milk [10].

Therefore, this study aimed to investigate the presence of tetracycline (oxytetracycline, tetracycline, and chlortetracycline) residues in fresh dairy milk collected from smallholder farms in the districts of Malang, Boyolali, and Padang Panjang, Indonesia, and to estimate the dietary exposure of tetracycline residues through milk consumption in children (10–12 years old) and adults (>18 years old).

## Materials and Methods

### Ethical approval

This study does not require ethical approval. This study was conducted with permission from Districts Livestock Services staff in Boyolali (Central Java province), Malang (East Java province), and Padang Panjang (West Sumatra province).

### Study period and location and sample collection

A cross-sectional survey was conducted in Indonesia, in Malang district (East Java province) and Boyolali district (Central Java Province) for one week in April 2018 and Padang Panjang district (West Sumatera Province) also for one week in August 2018. A total of 203 fresh dairy milk samples (roughly 200 mL per sample) was collected randomly from individual lactation dairy cows with no lactation age limitation, namely 91, 47, and 66 samples were from Malang (East Java province), Boyolali (central Java province), and Padang Panjang (West Sumatera province), respectively. The milk samples were frozen in plastic bottles and brought to the laboratory. Subsequently, all samples were stored at -20°C until further examination at the Toxicology Laboratory, Research Institute for Veterinary Science, Bogor (West Java Province), Indonesia.

### Chemicals and equipment

Analytical-grade chemicals from Merck (Darmstadt, Germany) were used. Ultrapure water was obtained from Milli Q direct 8/16 system (Millipore SAS, 67120 Molsheim, France). High-purity tetracycline standards (oxytetracycline, tetracycline, and chlortetracycline) were supplied by Vetranal (Sigma-Aldrich, Darmstadt, Germany). Stock standard solutions of 1000 ppm were prepared in methanol and stored at −20°C. Working standard solutions were prepared by diluting the stock standard solution in methanol. McIlvaine buffer solution was prepared by dissolving 11.8 g of citric acid monohydrate, 13.72 g of disodium hydrogen phosphate dihydrate, and 33.62 g of ethylene diamine-tetra acetic acid disodium salt in 1 L of water.

### Sample extraction and analysis

Sample extraction was performed using the method described by Cinquina *et al*. [11]. Briefly, 5 g of thawed milk sample was mixed with 2 mL of 20% trichloroacetic acid in a 50 mL centrifuge propylene tube, mixed and shaken with 15 mL of McIlvaine buffer, centrifuged for 15 min at 3461× *g*, and filtered. The supernatant was removed and a second extraction step was performed. The combined supernatant was passed through a Bond-Elut C18 solid phase extraction cartridge (methanol-activated, water, and McIlvaine buffer), eluted with methanol, and dried in a nitrogen stream at 30°C. The residue was diluted in 1 mL of the mobile phase, filtered through a 0.45-μm nylon filter, and injected into a Shimadzu LC-20AD HPLC- photo diode array (355 and 368 nm). The mobile phase was prepared using 0.0025M oxalic acid and acetonitrile (4:1 v/v), and run isocratic at 1.0 mL/min.

In-house method for partial validation was used [9] to estimate the recovery rate and limit of detection (LOD). The results are summarized as follows: Recovery rates were 92.54%, 80.83%, and 80.65% and LODs were 5.9, 5.3, and 13.5 ng/g for oxytetracycline, tetracycline, and chlortetracycline, respectively. Samples with values above or below LOD were considered positive or undetected, respectively.

### Assessment of dietary exposure to tetracycline residues

Risk assessment uses the hazard quotient (HQ) to indicate the ratio of a substance’s exposure concentration and the level at which there are no harmful effects on human health. Calculating HQ involves comparing estimated daily intake (EDI) to ADI recommended by JECFA (HQ = EDI/ADI) [12]. If HQ ≤1, the risk to human health exposure is not significant; if HQ is >1, the consumer is at risk. Estimated daily intake was calculated using the following equation [13]:


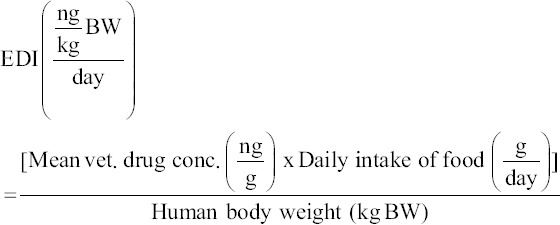


The daily intake of milk in Indonesia is 45.4 g/day [14] and the BW of adults (>18 years old) and children (10–12 years old) are 60 kg and 36 kg, respectively [15].

## Results

### Antibiotic residues in milk samples

Oxytetracycline, tetracycline, and chlortetracycline were found in milk from almost all locations, except Boyolali, which showed absence of chlortetracycline (non-detectable or below LOD) (Table-1). The prevalence rates of milk samples contaminated with tetracycline residues were <10%. Chlortetracycline was the most abundant (8.37%; mean 137.05 ± 132.70 ng/g), followed by tetracycline (7.88%; mean 21.76 ± 10.47 ng/g) and oxytetracycline (5.91%; mean 52.20 ± 20.30 ng/g). Seven (3.45%) samples exceeded the MRL of chlortetracycline (highest concentration of 498.4 ng/g), but no sample exceeded the MRL for oxytetracycline and tetracycline.

**Table-1:** The prevalence and residue concentration of tetracyclines in milk samples from three districts.

Antibiotics	Districts	n	Positive samples				n (%) samples ≥MRL

			n	%	Concentration (ng/g)		

					Range	Mean ± standard deviation	
Oxytetracycline	Malang	91	2	2.19	24.5–85.6	55.05 ± 43.20	Nil
	Boyolali	47	1	2.13	48.10	48.10 ± 0.00	Nil
	Padang Panjang	66	9	24.24	11.6–76.4	52.02 ± 27.12	Nil
	Total	203	12	5.91	11.6–85.6	52.20 ± 20.30	Nil
Tetracycline	Malang	91	6	6.59	15.4–49.4	28.80 ± 11.44	Nil
	Boyolali	47	8	17.02	11.7–35.9	17.63 ± 8.09	Nil
	Padang Panjang	66	2	3.03	11.9–22.4	17.15 ± 7.42	Nil
	Total	203	16	7.88	11.7–49.4	21.76 ± 10.47	Nil
Chlortetracycline	Malang	91	16	17.58	14.8–498.4	134.49 ± 128.92	7 (7.69%)
	Boyolali	47	Nil	Nil	ND	ND	Nil
	Padang Panjang	66	1	1.51	93.50	93.50 ± 0.00	Nil
	Total	203	17	8.37	14.8–498.4	137.05 ± 132.70	7 (3.45%)

MRL=100 ng/g, ND=Non-detected, MRL=Maximum residue limit

Dietary exposure to tetracycline through consumption of even very small amounts of milk (EDI) by children (10–12 years old) and adults (>18 years old) was assessed using HQ (Table-2) [5]. In children, chlortetracycline (172.83 ng/kg BW/day) had the highest observed EDI value, followed by oxytetracycline (65.83 ng/kg BW/day) and tetracycline (27.44 ng/kg BW/day). Therefore, chlortetracycline had the highest observed HQ value (0.0058), whereas oxytetracycline had the lowest HQ value (0.0009). Consistently, in adults, chlortetracycline (103.70 ng/kg BW/day) had the highest EDI value, followed by oxytetracycline (39.50 ng/kg BW/day) and tetracycline (16.46 ng/kg BW/day). Therefore, chlortetracycline had the highest HQ value (0.0035) and oxytetracycline had the lowest HQ value (0.0006). Because HQ values for antibiotic residues found in milk samples from dairy farms were <1 (not exceeding the recommended ADI limit), the correlation between milk consumption and health effects both in children and adults was negligible.

**Table-2:** EDI and HQ values of tetracycline residue through milk consumption on children (10–12 years) and adult (>18 years) population in Indonesia.

Antibiotics	Population	EDI (ng/kg BW/day)	Acceptable daily intake (ng/kg BW/day) [5]	HQ
Oxytetracycline	Children	65.83	30.000	0.0022
	Adult	39.50		0.0033
Tetracycline	Children	27.44	30.000	0.0009
	Adult	16.46		0.0006
Chlortetracycline	Children	172.83	30.000	0.0058
	Adult	103.70		0.0035

EDI=Estimated dietary exposure, HQ=Hazard quotient, BW=Body weight

## Discussion

Milk that contains antibiotic residues is not suitable for human consumption. Several field studies using various techniques have been conducted to investigate antibiotic residues in milk [16]. The findings suggest that several farmers in Indonesia were using three types of tetracycline as veterinary drugs. Low prevalence of antibiotic residues obtained in this study could be attributed to factors such as observation of withdrawal time and implementation of good veterinary practice, except for 7 (3.45%) samples collected in Malang district that exceeded the MRL. These samples might be lacking in the observation of withdrawal period, as was observed with farmers in Niger [17].

Antibacterial residues in milk and milk products are common in many countries. This finding is comparable with the results found in Punjab, India [18], where 18/133 (13.5%) milk samples contained 16–134.5 ng/g tetracycline, and oxytetracycline showed the highest incidence (86%) of detected contamination compared with other tetracyclines. Another study from India showed 62.7–177 ng/g oxytetracycline in 8/164 samples and 72.6–85.1 ng/g tetracycline in 2/164 samples [11]. In Ethiopia, 48/400 (12%) samples were positive for oxytetracycline, with a mean residue level of 125.25 ng/g [19]. Oxytetracycline, tetracycline, chlortetracycline, and doxycycline (range: 45.6–163.5 ng/g) were found in 8/90 (8.8%) raw milk samples from cows, goats, and sheep [20].

In contrast, the prevalence of tetracycline residues was low (3%) in 100 milk samples collected from the State of Paraná, Brazil – oxytetracycline (121.8 ng/g), tetracycline (93.5 ng/g), and chlortetracycline (61.6 ng/g) [21]. Consistently, in China [22], the prevalence was very low –1.10% for tetracycline and 0.55% for oxytetracycline in 182 pasteurized samples from milk bars, with maximum residue levels of 11.9 ng/g and 26.9 ng/g, respectively, and even negative for 229 milk samples from Kenya [23].

Antibiotics used to treat intramammary infection are present in cows at the time of milking, and therefore, it was suggested that cows treated with antibiotics should not be milked until the antibiotic has been completely removed [24]. The withdrawal time of oxytetracycline in milk is 96 h after the last treatment [25]. In the case of mastitis treatment, older cows were slower to eliminate tetracycline [26]. Therefore, consuming milk from an animal treated with tetracycline and developing subclinical mastitis should be restricted to at least 4 days after milk discharge [27]. High dosages and disregard for grace periods while using antibiotics in dairy production could endanger public health and cause financial loss for the dairy industry, because antibiotics are stable during pasteurization – only 5.74% and 15.3% of tetracycline and oxytetracycline decreased, respectively [28]. Therefore, to reduce the risk of antibiotic residues in milk, it is critical to adhere to antibiotic withdrawal time [29].

Another concern with improper use of antibiotics is the emergence of contamination released in the environment in active form, because >70% of tetracycline antibiotics are excreted by both humans and animals [30]. Tetracyclines are stable in the environment and difficult to oxidize, although they are unstable at acidic pH [22], forming epi- and anhydro-products [31].

Our study on risk assessment in Indonesian adults showed lower HQ values than those from the study in Punjab, India [18], which showed HQ values of 0.027 and 0.03 for tetracycline and oxytetracycline residues in pooled raw milk samples, respectively. Very low HQ values were obtained for adult consumers in India – 0.009 for oxytetracycline, 0.004 for tetracycline, and 0.002 for chlortetracycline [32]. A study in Bangladesh revealed an HQ of 0.0056 for tetracycline residues in 5 (3.33%) positive milk samples [33]. A study of 173 milk samples from a northwestern Himalayan state of India showed hazard indices of 0.092 and 0.047 for oxytetracycline in raw and pasteurized milk, respectively [34]. Long-term consumption by children (BW of 15 kg) can expose them to more risk. Oxytetracycline, which was found in 23/252 pasteurized milk samples in Brazil, resulted in the EDI being equal to or lower than the maximum recommended ADI by the European Union [35]. An EDI of 0.05 g/kg BW/day or 1% of the permitted daily intake of pasteurized cow milk in Brazil indicated that the danger to the public from these residues in milk was negligible [21]. The EDI of tetracycline residues was found to be 1.28 ng/kg BW/day for raw milk and 2.09 ng/kg BW/day for total dairy products in Lebanon [36].

The size of the dairy farm did not contribute to differences in HQ values for oxytetracycline or tetracycline in adults or children in Punjab, India [13]. In Iran, different age groups (children and adults) were associated with varied risk characterizations of dietary exposure to tetracycline residues through milk consumption, because the consumers drank milk 2 times per day (480 mL), that is, 7%–30% of ADI (equal to HQs of 0.0007–0.003) [3].

Nevertheless, low amounts of antibiotics may be detrimental to human health, even if all EDI values for tetracyclines in this study were low in comparison to ADI. In addition, long-term effects on the consumption of other contaminated animal products should be considered [23]. Notably, the effect of even very low concentrations of one antibiotic or a combination of two or more antibiotics, especially with heavy metals, can lead to antibiotic resistance [37]. Conventional cooking methods are unable to destroy all veterinary drug residues in foods [38]. Therefore, a control program for monitoring milk safety is necessary.

## Conclusion

This study found three types of tetracycline residues (oxytetracycline, tetracycline, and chlortetracycline) in almost all milk sampling locations, except for Boyolali, which lacked chlortetracycline. The highest prevalence and residue concentration were obtained for chlortetracycline, some of which even exceeded the MRL. The presence of antibiotic residues in milk from several individual farms shows that some farmers did not observe the withdrawal times in lactating animals, especially when concentrations were higher than the MRL. However, the EDIs of tetracycline consumed through milk by 10–12 years old and adults were low and the risks to consumers were negligible. Nevertheless, this study emphasizes the importance of routine monitoring of antibiotics and awareness programs for farmers to minimize the use of antibiotics and respect the withdrawal times of antimicrobials to produce safe food.

## Authors’ Contributions

RW: Designed the study, collected samples, data analysis, and drafted the manuscript. EM: Collected the data, designed the study, statistical analysis, and reviewed and edited the manuscript. YA: Processed the samples and reviewed and edited the manuscript. All authors have read, reviewed, and approved the final manuscript.
